# Chondrocalcinosis and the haemochromatosis-linked *HFE* C282Y homozygous variant in the UK Biobank

**DOI:** 10.1016/j.ero.2025.11.016

**Published:** 2025-12-15

**Authors:** Lucy R Banfield, Karen M Knapp, Luke C Pilling, David Melzer, Janice L Atkins

**Affiliations:** 1 The Department of Health and Care Professions, Faculty of Health and Life Sciences, University of Exeter, St Luke’s Campus, Exeter, UK; 2 The Department of Clinical and Biomedical Sciences, Faculty of Health and Life Sciences, University of Exeter, St Luke’s Campus, Exeter, UK; 3 Emeritus Professor (retired)*,* University of Exeter, St Luke’s Campus, Exeter, UK

## Abstract

**Objectives:**

C282Y genetic homozygosity is the main cause of the iron-overload disorder haemochromatosis. Musculoskeletal pain and arthropathy are common in haemochromatosis, but less is known about chondrocalcinosis (cartilage calcification) with the C282Y variant, especially in the community. We assessed knee chondrocalcinosis in intelligent dual-energy X-ray absorptiometer (iDXA) images from UK Biobank volunteers by *HFE* genotype.

**Methods:**

Data were from 236 European genetic ancestry C282Y homozygotes and 236 age, sex, and body mass index (BMI)-matched controls with no C282Y alleles (48-80 years, mean 64.6, SD ±7.6). Of 472 participants, 435 had relevant left and right knee iDXA imaging. Evidence of chondrocalcinosis was assessed by an experienced reporting radiographer blind to genotype to ensure unbiased and objective evaluation. Logistic regression models were age, sex, and BMI matched.

**Results:**

C282Y homozygotes had significantly increased odds of knee chondrocalcinosis (odds ratio [OR]: 3.79, 95% CI: 1.51-9.55, *P* = .005). Among males, 15.9% (14/88) of homozygotes showed chondrocalcinosis vs <5.9% (<5/84) of controls (OR = 7.76, 95% CI: 1.71-35.25, *P* = .008). Of affected males, 57.1% reported knee pain, yet fewer than 28.6% had a haemochromatosis diagnosis. In females, 6.0% (8/134) of homozygotes had chondrocalcinosis vs <3.9% (<5/129) of controls, but the association was not statistically significant (OR = 1.98, 95% CI: 0.58-6.76, *P* = .27), suggesting a need for larger samples.

**Conclusions:**

In this community-genotyped sample, male C282Y homozygotes had markedly increased odds of knee chondrocalcinosis. Evaluation of serum ferritin levels to identify possible haemochromatosis may be justified in knee chondrocalcinosis management.

**WHAT IS ALREADY KNOWN ON THIS TOPIC**

• Chondrocalcinosis is a characteristic manifestation of haemochromatosis and a defining feature of its associated arthropathy.

**WHAT THIS STUDY ADDS**

• Chondrocalcinosis can act as an early radiological marker of haemochromatosis in male C282Y homozygotes, preceding formal clinical diagnosis.

**HOW THIS STUDY MIGHT AFFECT RESEARCH, PRACTICE OR POLICY**

• Recognition of chondrocalcinosis in imaging, with the absence of coexisting degenerative disease, should prompt earlier investigation for haemochromatosis, facilitating timely detection and intervention for iron overload.

Alt-text: Unlabelled box dummy alt text

## INTRODUCTION

Haemochromatosis is an autosomal recessive condition resulting in iron overload predominantly caused by mutations in the *HFE* gene, in particular the C282Y mutation and to a lesser extent the H63D mutation [1]. C282Y homozygosity is the mutation primarily associated with haemochromatosis, compared to other lower penetrance C282Y/H63D genotypes [1]. Consequently, for the purpose of this study, we elected to focus on C282Y homozygotes.

This excessive absorption of iron, associated with haemochromatosis, and its subsequent accumulation can have a detrimental impact on several organs. The clinical disease has been associated with osteoarthritis (OA), diabetes, liver disease, including liver cancer, as well as several other conditions [[2], [3], [4]]. Despite being the most common genetic disease in those of Northern European ancestry, diagnoses of haemochromatosis may be missed or delayed, with only a minority of community populations with the C282Y homozygote variant being diagnosed [5].

Several musculoskeletal complications are associated with haemochromatosis in individuals with C282Y homozygosity, and particularly among males. A number of previous studies have identified an increased risk of joint replacement surgeries [[5], [6], [7], [8]] osteoporosis and fracture [[9], [10], [11], [12]]. The arthritis associated with haemochromatosis has been a well-recognised component of the disease for several years, with findings typically described as affecting primarily the metacarpophalangeal joints, the ankle, knee, and hip, as well as other less typical joints, such as the shoulders and wrists [[13], [14], [15]]. Clinical presentation may include pain, swelling, stiffness, and a limited range of motion, accompanied by exuberant osteophyte formation. These findings can often be bilateral and are reported to occur in two-thirds of those with haemochromatosis [16,17].

The mechanisms underlying chondrocalcinosis (CC) in C282Y homozygotes remain incompletely understood. Although iron overload has been proposed as a contributing factor, it is important to recognise that the C282Y genotype itself affects hepcidin regulation, potentially leading to downstream effects independent of measurable iron overload. One hypothesis is that excessive haemosiderin deposition in the synovial membrane may trigger inflammation of synovial tissues and articular cartilage, contributing to joint degeneration. A study by Dejaco et al [18] found that inflammatory changes and synovitis were prevalent in those with haemochromatosis arthropathy, and that subclinical inflammation was also seen in the absence of haemochromatosis arthritis. This is supported by a recent study, which identified that iron overload may induce M1 polarisation in macrophages, resulting in the release of proinflammatory factors and joint degeneration [19]. Macrophages are one of the 2 main cellular components of synovium and serve to engulf pathogens and debris, relative to ‘normal’ tissue ageing, to maintain a healthy joint [20]. However, if abnormally activated, M1 macrophage proinflammatory cytokines are key drivers of chronic inflammation and can also contribute to tissue damage. Although this can be beneficial in the short term when fighting infections, it can be detrimental in the long term.

With regard to CC (cartilage calcification), there is wide variation in reported frequency among patients with haemochromatosis, with estimates ranging from 5% to 49% [[21], [22], [23]]. In a small study of 18 patients with diagnosed haemochromatosis undergoing venesection treatment, CC prevalence increased from 39% to 72% over a 10-year follow-up [24]. However, less is known about the incidence of CC in those with the C282Y mutation. This study examines associations between CC in the knee and *HFE* C282Y homozygosity (compared with age-, sex-, and body mass index [BMI]-matched controls without C282Y alleles) within a subset of UK Biobank (UKB) community-dwelling participants.

## METHODS

### UKB participants

UKB is a cohort study of 502,150 UK adults aged 37 to 73 years at baseline assessment (2006-2010). Data include baseline characteristics, biomarkers, genetics, and linked medical records. UKB data are available to bona fide researchers upon application. The Northwest Multi-Centre Research Ethics Committee approved the collection and use of UKB data (Research Ethics Committee reference 11/NW/0382). Participants provided informed consent for the use of their data, health records, and biological materials for health-related research. Access to UKB was granted under application number 14631. To protect participant confidentiality, the UKB requires that data or aggregate statistics corresponding to fewer than 5 participants cannot be published, nor can data that allow a participant count of less than 5 to be derived [25].

### Genotype data

Genotype information on *HFE* C282Y (rs1800562 A allele) and *HFE* H63D (rs1799945 G allele) was from whole exome sequencing data (methods by Regeneron [26]). Patient consent did not include feedback to patients on genotypes. We included 451,270 participants genetically similar to the 1000 Genome Project European Ancestry superpopulation (EUR-like’ [27]), of which 2902 (0.64%) were C282Y homozygotes.

### Intelligent dual-energy X-ray absorptiometer data

**Figure 1:**
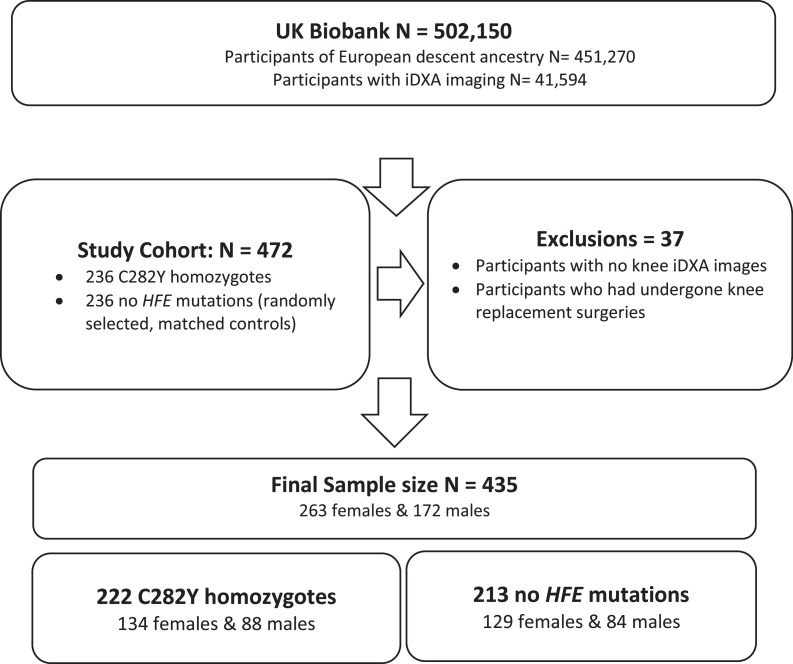
Flowchart of included/excluded participants. iDXA, intelligent DXA.

**Figure 2:**
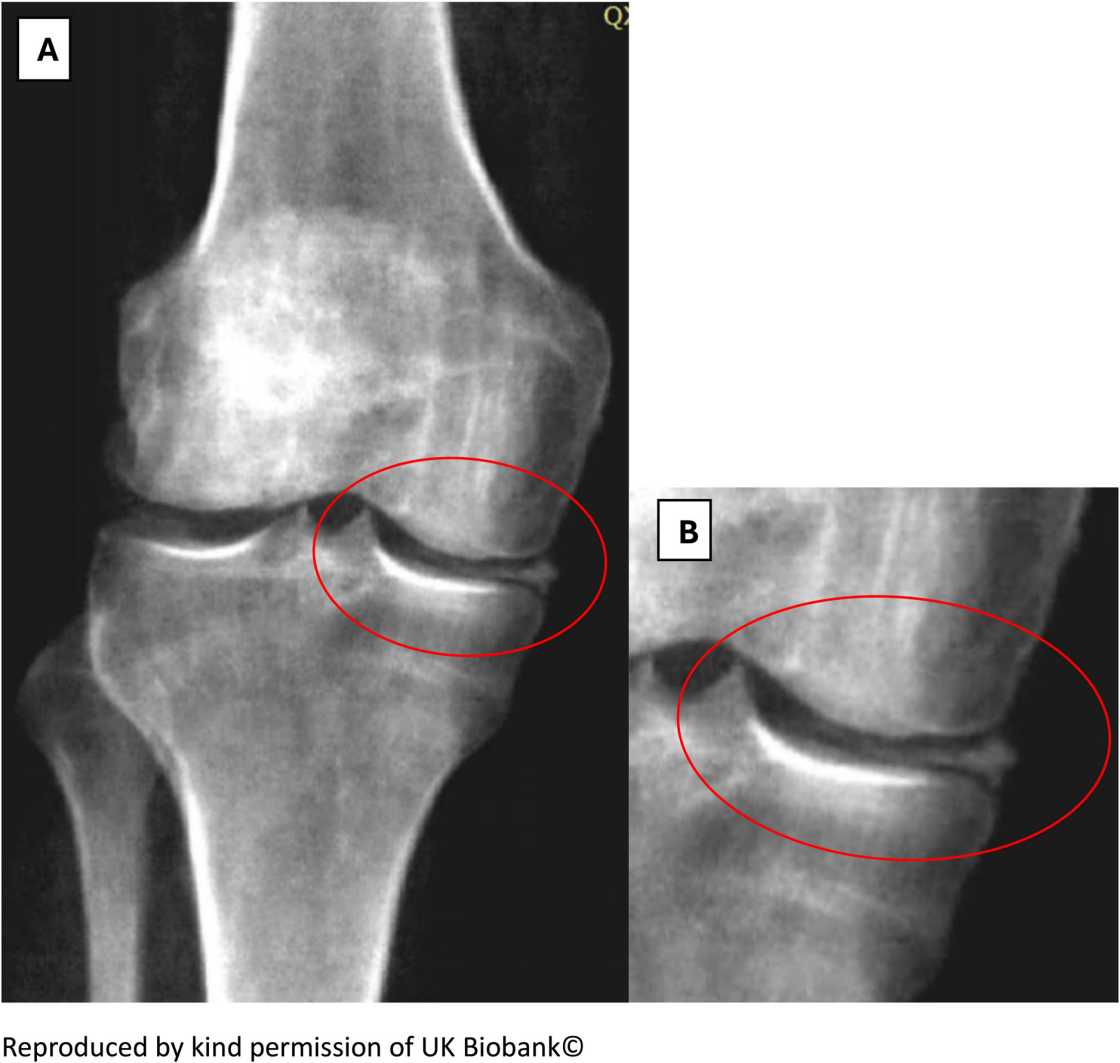
Example of chondrocalcinosis of the knee on an iDXA image in UK Biobank (A) with an enlarged picture (B) to highlight the presence of chondrocalcinosis in the medial tibiofemoral space. Reproduced by kind permission of UK Biob ank©. iDXA, intelligent dual-energy X-ray absorptiometer.

During follow-up, a subset of UKB participants (n = 41,594/451,270 at the time of analysis) attended a follow-up visit for intelligent dual-energy X-ray absorptiometer (iDXA) imaging (2014 to 2020, aged 45-82 years). As part of a suite of imaging examinations, iDXA high-resolution imaging of each knee was performed using a GE Lunar [28]. Within this iDXA subset, we used all available data from 236 C282Y homozygotes, along with 236 age, self-reported sex, and BMI-matched controls with no C282Y or H63D genotypes (n = 472). Within these 472 participants, 435 had relevant right and left knee iDXA imaging (263 females and 172 males) (see Fig 1 for flowchart of included participants). A small number of this subset had undergone knee replacement surgery before imaging, which excluded those joints from iDXA. Others were excluded due to poor image quality or missing images. These iDXA knee images were reviewed for radiological evidence of CC within the tibiofemoral joint space by an experienced reporting radiographer (LRB), blind to participant genotype, to ensure unbiased and objective evaluation. CC was classified if present in either the left or right knee (see Fig 2).

We excluded participants who had ever been diagnosed with rheumatoid arthritis, multiple myeloma, malignant neoplasm or secondary malignant neoplasm of bone, or benign neoplasm of bone (including diagnoses from baseline self-report and hospital inpatient follow-up until 2022) (see Table 1 for coding used).

**Table 1:** Incident disease coding from follow-up in hospital inpatient data

Disease/procedure	ICD-10 codes
Rheumatoid arthritis	M05, M05.8; M05.9; M06.0; M06.8
Multiple myeloma	C90.0; C90.0; C90.2; C41.0 C90.3
Malignant neoplasm of bone	C40; C40.1; C40.2; C40.3; C40.8; C40.9; C41.0; C41.1; C41.2; C41.3; C41.4; C41.8; C41.
Secondary malignant neoplasm of bone	C79.5
Benign neoplasm of bone	D16.0; D16.1; D16.2; D16.4; D16.5; D16.6; D16.7; D16.8; D16.9

ICD-10, International Classification of Diseases 10th revision codes.

### Additional variables (at the time of the iDXA)

Participants were asked to self-report if they had knee pain that lasted more than 3 months. The prevalence of haemochromatosis and OA was based on diagnoses from baseline self-reports plus hospital inpatient data from 1996 to the date of the iDXA visit, as coded previously [7].

### Statistical analysis

All analyses were carried out in STATA 18.0 using a matched analysis. *HFE* C282Y homozygotes were matched to controls (without C282Y or H63D alleles) for sex, age, and BMI category at time of dual-energy X-ray absorptiometer (DXA): underweight (<18.5 kg/m^2^), normal (18.5-24.9 kg/m^2^), overweight (25-29.9 kg/m^2^) or obese (≥30 kg/m^2^). We used random-effects logistic regression models to estimate associations between genotype and the likelihood of knee CC using the STATA function ‘xtlogit’, specifying pairwise matching as the random intercept to account for the within-pair correlation. This approach was chosen because our study used a matched case-control design. The xtlogit model is particularly suitable for analysing matched pair data with binary outcomes, effectively accounting for within-subject correlation and unobserved heterogeneity through random-effects models [29]. We performed random-effects logistic regression in all participants and stratified by sex. In sensitivity analysis, we tested for an interaction between sex and genotype for the odds of CC.

We examined the prevalence of chronic knee pain and OA within participants by genotype group and the presence of CC. We also performed additional sensitivity analysis, excluding those diagnosed with haemochromatosis at the time of the iDXA imaging.

## RESULTS

### Characteristics of participants

Analyses included 435 European descent participants aged 48 to 80 years at the time of iDXA scanning, 60.5% of the sample were women (n = 263), and the mean age of the cohort was 64.6 years (SD ± 7.6). There were 222 C282Y homozygotes (134 females and 88 males) (Table 2).

**Table 2:** UK Biobank participant characteristics of C282Y homozygotes with available iDXA data and randomly selected matched controls, with no *HFE* C282Y or H63D alleles^a^

Group	*HFE* genotype	Mean age, y (SD)	Haemochromatosis diagnosis at time of DXA (%)	OA at time of DXA (%)	Knee pain for 3+ mo (%)
Males					
No CC	No C282Y or H63D alleles	64.2 (8.0)	0 (0)	8 (9.8)	8 (9.8)
	C282Y homozygotes	64.1 (8.0)	20 (27.0)	<5 (<6.8)	13 (17.6)
CC present	No C282Y or H63D alleles	72.5 (3.5)	0 (0)	0 (0)	<5 (<50.1)
	C282Y homozygotes	66.6 (7.3)	<5 (<35.7)	0 (0)	8 (57.1)
Females					
No CC	No C282Y or H63D alleles	64.4 (7.3)	0 (0)	14 (11.2)	15 (12.0)
	C282Y homozygotes	64.6 (7.6)	12 (9.5)	10 (7.9)	23 (18.3)
CC present	No C282Y or H63D alleles	73.5 (4.5)	0 (0)	0 (0)	0 (0)
	C282Y homozygotes	68.0 (7.4)	0 (0)	0 (0)	<5 (<62.5)

BMI, body mass index; CC, chondrocalcinosis; DXA, dual-energy X-ray absorptiometer; iDXA, intelligent dual-energy X-ray absorptiometer; OA, osteoarthritis.

The ‘<’ symbol is used in accordance with UK Biobank’s data disclosure policy, which prohibits reporting exact counts below 5 to protect participant confidentiality.

a Controls matched on age, sex, and BMI category.

Overall, CC was present in either (or both) knees of 28 (6.4%) participants, 22 of whom were C282Y homozygotes. In males, knee CC was present in 15.9% of C282Y homozygotes (14/88) and <5.9% without *HFE* variants (<5/84). In females, knee CC was present in 6.0% of C282Y homozygotes (8/134) and <3.9% without *HFE* variants (<5/129). The mean age of those with CC was lower for both the male and female C282Y homozygotes (66.6 years and 68.0 years, respectively) when compared to matched controls with no *HFE* mutations (72.5 years and 73.5 years).

Matched regression analysis demonstrated significantly increased odds of CC in the C282Y homozygotes (odds ratio [OR]: 3.79, 95% CIs: 1.51-9.55, *P* = .005), compared to age, sex and BMI-matched controls without *HFE* C282Y or H63D alleles. The same analysis, stratifying by sex, demonstrated increased odds for male homozygotes (OR: 7.76, 95% CI: 1.71-35.25, *P* = .008), and although a modest increase in odds was also seen in female homozygotes, this was not significant (OR: 1.98, 95% CI: 0.58-6.76, *P* = .27) (Table 3). Although there was a difference in the prevalence of CC between males and females, sensitivity analysis showed no significant interaction between C282Y genotype and sex for odds of CC (*P* = .17).

**Table 3:** Increased odds of chondrocalcinosis in HFE C282Y homozygotes compared to controls^a^

Group	*HFE* genotype	N	CC (%)	OR	95% CIs	*P* value
Males	No C282Y or H63D alleles	84	<5 (<5.9)	1.00		
	C282Y homozygotes	88	14 (15.9)	7.76	1.71-35.25	.008
Females	No C282Y or H63D alleles	129	<5 (<3.9)	1.00		
	C282Y homozygotes	134	8 (6.0)	1.98	0.58-6.76	.27
All	No C282Y or H63D alleles	213	6 (2.8)	1.00		
	C282Y homozygotes	222	22 (9.9)	3.79	1.51-9.55	.005
	*Total*	435	28 (6.4)			

BMI, body mass index; CC, chondrocalcinosis; OR, odds ratio.

The ‘<’ symbol is used in accordance with UK Biobank’s data disclosure policy, which prohibits reporting exact counts below 5 to protect participant confidentiality.

a Controls with no *HFE* C282Y or H63D alleles, matched on age, sex, and BMI category.

### Sensitivity analyses (excluding haemochromatosis diagnosis)

To determine whether clinical haemochromatosis impacted CC prevalence within this subset, we excluded those who had been diagnosed with haemochromatosis at the time of iDXA imaging. Within our dataset, there were 36 homozygotes with a haemochromatosis diagnosis (n = 24 male, 27.3%, and n = 12 female, 9.0%). When those with a haemochromatosis diagnosis at the time of imaging (n = 36) were excluded, a higher proportion of CC was still evident in both male (n = 10, 15.6%) and female (n = 8, 6.6%) homozygotes when compared to the wildtype male (<5.9%) and female (<3.9%) participants. This increased risk remained high for male homozygotes who had not been diagnosed with haemochromatosis at the time of imaging (OR = 7.59, 95% CI: 1.60-35.99, *P* = .011), but again, although increased odds were seen in the female homozygotes, this was not significant (OR = 2.19, 95% CI: 0.64-7.48, *P* = .210) (Table 4).

**Table 4:** Increased odds of chondrocalcinosis in HFE C282Y homozygotes compared to controls, excluding those with diagnosed haemochromatosis^a^

Group	*HFE* genotype	N	CC (%)	OR	95% CIs	*P* value
Males	No C282Y or H63D alleles	84	<5 (<5.9)	1.00		
	C282Y homozygotes	64	10 (15.6)	7.59	1.60-35.99	.01
Females	No C282Y or H63D alleles	129	<5 (<3.9)	1.00		
	C282Y homozygotes	122	8 (6.6)	2.19	0.64-7.48	.21

BMI, body mass index; CC, chondrocalcinosis; OR, odds ratio.

The ‘<’ symbol is used in accordance with UK Biobank’s data disclosure policy, which prohibits reporting exact counts below 5 to protect participant confidentiality.

a Controls with no *HFE* C282Y or H63D alleles matched on age, sex, and BMI category.

### OA and joint replacement associations

The prevalence of OA at the time of DXA imaging was slightly higher in those without *HFE* mutations (12 homozygotes vs 22 wildtypes). Of these, a greater number was observed in female (n = 10) homozygotes compared to male (n = <5) homozygotes. No homozygous participants had concurrent OA and CC at the time of imaging.

### Knee pain

When investigating the association between ‘knee pain for 3 + months’ and the presence of CC, we again found reported pain and CC to be more prevalent among male homozygotes (n = 8, 57.1%) compared to those without the *HFE* mutation (<50%). Conversely, the number of female participants with both CC and reported ‘knee pain for 3+ months’ was notably lower, with only 1 female homozygote and no wildtype females affected (Table 2). Excluding individuals diagnosed with haemochromatosis reduced the number of male homozygotes with both pain and CC further.

## DISCUSSION

We used a subset of participants from the UKB cohort study (the largest community sample of haemochromatosis-genotype individuals to date) to examine associations between *HFE* C282Y homozygosity and the presence of CC within the tibiofemoral joint as identified on iDXA imaging. We specifically focused on high-risk C282Y homozygotes in this study, excluding other *HFE*-related mutations, such as H63D homozygotes and C282Y-H63D compound heterozygotes. We compared C282Y homozygotes with age-, sex-, and BMI-matched controls without *HFE* genotypes. Our analysis revealed a higher incidence of CC in the knee joints of both male and female C282Y homozygotes compared to matched wildtypes, with a notably elevated risk observed among male homozygotes. Although previous studies have reported an increased prevalence of CC associated with clinical haemochromatosis, our findings offer a more detailed understanding of the genotype-specific risk within a large community-based sample of predominantly undiagnosed individuals, and, to our knowledge, are the largest study to date on community-genotyped C282Y homozygotes and CC.

Our study found a prevalence of CC of 9.9% among individuals homozygous for the C282Y mutation, rising to 15.9% in male homozygotes. This is lower than the 24% prevalence of haemochromatosis-associated arthropathy reported by Carroll et al [22] in a cohort of clinically diagnosed patients with haemochromatosis, all of whom were also C282Y homozygotes. Their higher prevalence may reflect more advanced disease, as their cohort included individuals with significant iron overload, a known risk factor for joint pathology. Similarly, Huaux et al [23] reported a CC prevalence of 20% in a cohort of patients with confirmed haemochromatosis, with articular symptoms often preceding systemic diagnosis. The lower prevalence observed in our cohort may reflect earlier disease stages or underdiagnosis, as our cohort was defined genetically rather than clinically.

CC within the knee joint is commonly associated with degenerative changes and is frequently seen in conjunction with OA [30,31] and haemochromatosis [32]. A previous paper by Timms et al [33] recruited participants according to the presence of either CC or calcium pyrophosphate dihydrate disease and then subsequently assessed for associations via genotype and with control participants (n = 18 C282Y homozygotes). Although this analysis included a small sample of C282Y homozygotes and did not stratify by sex, it identified a connection between C282Y homozygosity and CC (relative risk 3.4, *P* = .037). Our findings further confirm the significant associations between homozygosity and tibiofemoral CC when compared to those with no *HFE* genetic mutations, and in addition highlight that the incidence of CC in male homozygotes is almost double that of the female homozygotes.

Our data analysis also identified that the mean age of those within our study who were C282Y homozygotes and had tibiofemoral CC was lower than that of the wildtypes. This is consistent with other studies examining joint changes associated with haemochromatosis [34,35]. Participants in these studies have usually been those with a haemochromatosis diagnosis; however, after excluding those in our cohort who had been diagnosed at the time of iDXA, we still observed a lower mean age in C282Y homozygotes.

These findings suggest that CC in this context may be more closely associated with the presence of the *HFE* mutation itself, potentially through metabolic or regulatory mechanisms—such as altered hepcidin activity—rather than being solely attributable to cumulative iron loading, particularly given that iron status in these participants is unknown. In contrast, CC observed in individuals without *HFE* mutations is more likely to reflect age-related changes and degenerative joint disease. This interpretation may be further supported by the apparent lack of OA diagnoses among homozygotes with CC in our cohort. However, it is important to note that OA data were derived from hospital records and did not include primary care sources, so OA prevalence at the time of imaging may be underreported. Haemochromatosis-related complications, including diabetes and infections, have also been observed in individuals with C282Y homozygosity, even when transferrin saturation and ferritin levels are within normal ranges [36], [37]. This further supports the possibility that mechanisms beyond iron overload—such as hepcidin dysregulation or other genotype-related effects—may contribute to the pathogenesis of CC. Further research is needed to disentangle these pathways and clarify the biological basis of joint degeneration in this population.

Chronic pain is another characteristic associated with CC, haemochromatosis, and C282Y homozygosity [38,39] and although we saw increased numbers of male and female homozygotes reporting knee pain, when we assessed these outcomes against the presence of CC, we found no significant increased associations between pain, homozygosity, and the presence of CC.

These findings raise important considerations regarding the clinical implications of C282Y homozygosity, adding to the ongoing debate around population screening for haemochromatosis. Although a national screening programme is not currently implemented in the UK [40], primarily due to concerns about low clinical penetrance, the potential for genotype-associated complications may warrant reconsideration of targeted screening approaches in older adults. Recent parliamentary reviews have upheld the decision not to introduce routine screening [41]; however, our findings support the need for further evaluation of age- and risk-based strategies to improve early identification and management of haemochromatosis-related complications.

### Strengths and limitations

This study has several strengths. It is the largest cohort of community-genotyped C282Y homozygotes, with iDXA data for the assessment of CC to date. It has provided robust data, including imaging, self-reports, and hospital records, to evaluate several factors that may contribute to or initiate CC in the knee joint. The images were reviewed by an experienced reporting radiographer who was blind to the genotype. However, there are some limitations to consider. First, UKB participants were healthier than the general population at baseline [42] and there is a slight chance of misclassification bias for self-reported disease outcomes at baseline, but this is minimised by the fact that a trained nurse conducted the interviews with participants. Second, no data were available on ferritin concentrations or transferrin saturation, so we are unable to examine iron loading within genotype groups. Third, disease diagnosis may be underestimated, as primary care data were not used in the analysis because those records were available only for a subset of this cohort. Lastly, we studied a European ancestry population, so results may not be generalisable to more diverse populations.

### Conclusion

In conclusion, these findings suggest that further investigation for iron overload, including serum ferritin and transferrin saturation levels, should be considered when CC is detected on imaging, especially in younger patients and those without concurrent degenerative joint disease. Such an approach may facilitate earlier identification of iron overload, allowing for timely implementation of appropriate haemochromatosis treatment regimens.

## CRediT authorship contribution statement

**Lucy R Banfield:** Writing – review & editing, Writing – original draft, Formal analysis, Conceptualization. **Karen M Knapp:** Writing – review & editing, Supervision. **Luke C Pilling:** Writing – review & editing, Supervision, Methodology, Formal analysis. **David Melzer:** Supervision, Conceptualization. **Janice L Atkins:** Writing – review & editing, Supervision, Conceptualization.

## Competing interests

All authors declare they have no competing interests.
